# Cortico-subthalamic connection predicts individual differences in value-driven choice bias

**DOI:** 10.1007/s00429-013-0561-3

**Published:** 2013-04-30

**Authors:** Martijn J. Mulder, Wouter Boekel, Roger Ratcliff, Birte U. Forstmann

**Affiliations:** 1 Cognitive Science Center Amsterdam, University of Amsterdam, Nieuwe Achtergracht 129, 1018 TV Amsterdam, The Netherlands; 2 Department of Psychology, Ohio State University, Columbus, OH USA

**Keywords:** Probabilistic tractography, vmPFC, STN, Perceptual decision making, Choice bias

## Abstract

It has been suggested that a connection between the STN and value-sensitive areas of the prefrontal cortex might mediate value-based actions in perceptual decision making. In this study, we first seek to quantify a structural connection between the STN and a cortical region that was associated with mechanisms underlying bias in choice behavior (vmPFC). Next, we tested whether individual differences in the probabilistic tract-strength of this connection were predictive for individual differences in the magnitude of bias in a perceptual decision-making task. Probabilistic tractography was used to measure the tract-strength between the STN and the vmPFC. Bias was quantified using an accumulation-to-bound model where a shift in the starting point of the accumulation of sensory evidence causes faster and more choices for an alternative that is more likely or more valuable. Results show that vmPFC is structurally connected with the STN and that the strength of this connection is predictive for choice bias towards an alternative that is more valuable, but not for choice bias towards an alternative that is more likely. These findings confirm the involvement of the cortico-subthalamic circuit in mechanisms underlying value-based actions in perceptual decision making.

## Introduction

The subthalamic nucleus (STN) has been implicated in mechanisms underlying perceptual decision making (Frank 2006; Fleming et al. 2010a, b; Simen et al. 2006; Bogacz and Gurney 2007; Bogacz 2007; Bogacz et al. 2010a; Cavanagh et al. 2011). These mechanisms are described by formal models that conceptualize the decision process as the accumulation of sensory information over time toward a decision threshold (for review, see Bogacz 2007; Gold and Shadlen 2007; Ratcliff and McKoon 2008; Wagenmakers 2009). The STN is believed to be involved in the adaptation of the decision threshold, which results in a change of distance between the starting and ending point of the accumulation process (Frank 2006). When the conflict between two response alternatives is high, an increase in the distance results in a longer time to decide, reducing premature choices.

Interestingly, evidence from human and animal studies has implicated the STN in value-based processes as well (Kantak et al. 2012; Matsumura et al. 1992; Baunez et al. 2002, 2005; Darbaky et al. 2005; Witjas et al. 2005; Teagarden and Rebec 2007; Lardeux and Baunez 2008; Lardeux et al. 2009; van Wouwe et al. 2011). Furthermore, computational models of perceptual decision making suggest that the STN might receive value-based information from cortical regions that are involved in processing value (e.g. OFC; Frank 2006). Along these lines, the STN might play a role in adjusting the distance between the starting and ending point of the accumulation process, which can be an optimal strategy to maximize reward (Bogacz et al. 2006; Feng et al. 2009; Simen et al. 2006, 2009). According to this idea, a preferred choice would correspond to a smaller distance than for the non-preferred choice, also giving rise to more and faster preferred choices (i.e., choice bias).

Bias mechanisms like these might be mediated by a connection between the STN and value-sensitive areas of the prefrontal cortex. Anatomical models have proposed a structural pathway originating from these cortical regions and the STN (Temel et al. 2005; Baunez and Lardeux 2011). Indeed, although cortical inputs to the STN seem to originate mainly from (pre)motor and lateral prefrontal cortex regions (Lambert et al. 2012; Aron et al. 2007; Aron and Poldrack 2006), evidence from animal studies show projections from the medial–orbital cortical areas to the STN in rodents (Maurice et al. 1998, 1999) and from the orbito/ventromedial areas to the STN in monkeys as well (Haynes and Haber 2013). Furthermore, it is shown that fibers originating from the monkey orbito/ventromedial area travel through the basal ganglia (Ferry et al. 2000; Haber et al. 2006) including the STN (Lehman et al. 2011). In line with these findings, studies using deep brain stimulation in patients with Parkinson’s disease reported changes in the blood flow and metabolism of orbito/ventromedial prefrontal regions (Brodmann area 11) after stimulation of the STN (Gjedde and Geday 2009; Kalbe et al. 2009). Although these studies suggest a connection between medial–orbital cortical areas and the STN, up until now it is unclear whether such structural pathway really exists in the human brain (Marani et al. 2008).

In this study, we first address the anatomical question whether there exists a structural connection between the STN and a value-sensitive region of the prefrontal cortex. For this purpose, we use diffusion weighted imaging (DWI) in combination with probabilistic tractography. As our cortical region, we choose a cluster (sphere) that is specifically associated with bias in perceptual decision making for the sample of subjects that are included in the current DWI analyses (vmPFC^1^ 1; Mulder et al. (2012). This region has been reported by studies of value-based processes in the orbito/ventromedial prefrontal cortex (e.g. Gläscher et al. 2009; Philiastides et al. 2010; Blair et al. 2006; Hampton et al. 2007; Smith et al. 2010; Liu et al. 2007; Ramnani et al. 2004; Knutson et al. 2004, 2005, 2007; Beckmann et al. 2009; Kable and Glimcher 2007). As such, this group-specific cortical cluster is a suitable candidate to test for an anatomical connection with the STN, as it is functionally associated with choice bias and value-based processes.

**Fig. 1:**
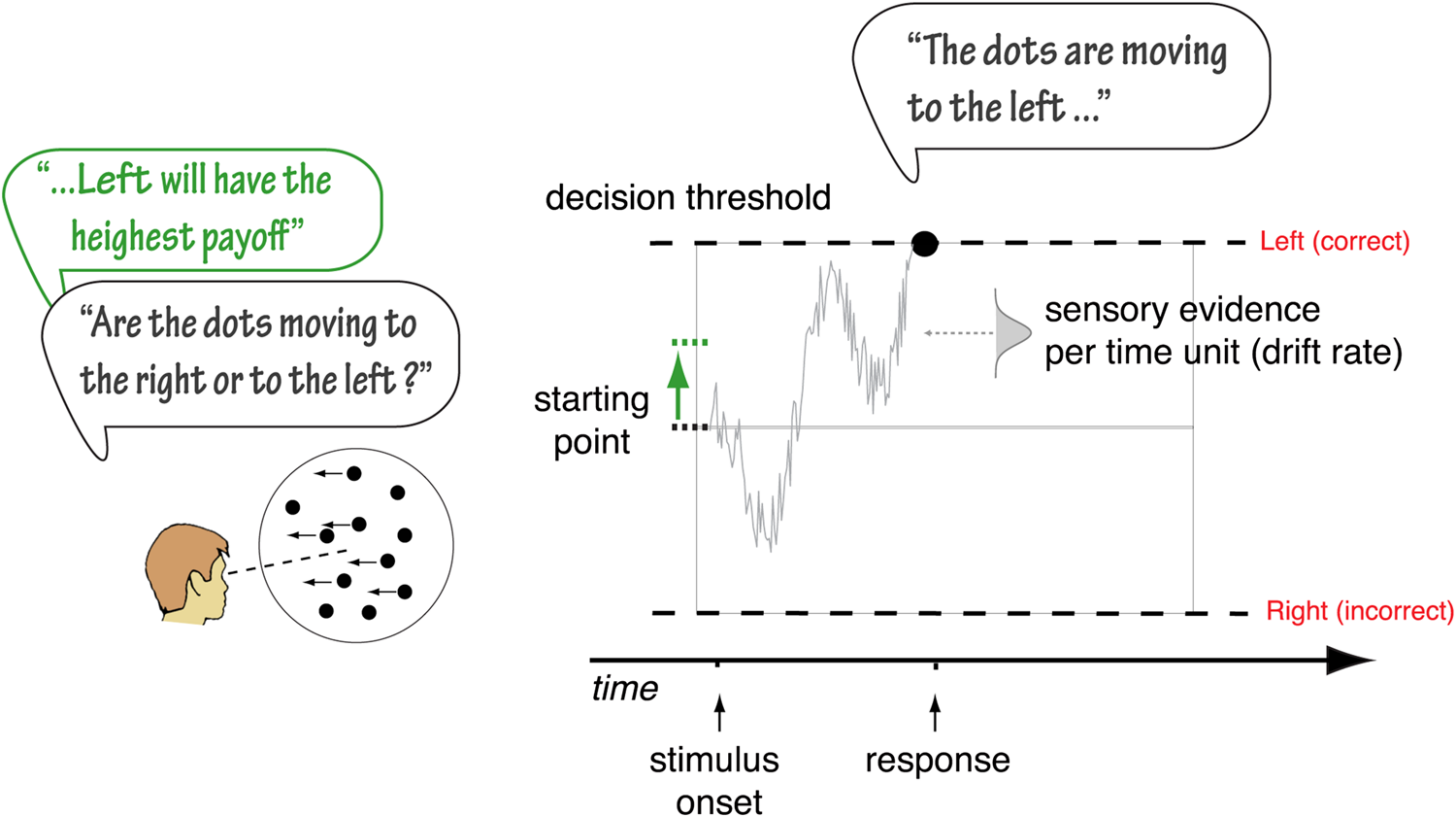
Schematic representation of the drift-diffusion model. The model assumes that dichotomous decisions are based on the accumulation of noisy evidence over time that starts at the starting point and ends at a decision threshold. Drift rate represents the average amount of evidence accumulated per time unit. Non-decision time is the time for processes other than the decision process. Prior information (*green text*) biases the decision process by adjusting the starting point, resulting in a smaller distance for the accumulation process to hit the decision threshold

Second, if such a connection exists, we will test whether individual differences in the strength of this connection predict individual differences in the magnitude of choice bias. Bias was measured using two versions of the random-dots motion task in which bias was manipulated by either assigning a larger reward to one of two alternatives (potential payoff) or changing the prior likelihood of occurrence for two alternatives (prior probability; Mulder et al. 2012). For each task, we fitted the drift-diffusion model (DDM; Ratcliff 1978) to the behavioral data and used the starting point parameter from the model to measure the magnitude of choice bias (Fig. 1). Importantly, the vmPFC region was associated with both types of bias (Mulder et al. 2012), allowing us to test whether the vmPFC–STN connection might be associated with mechanisms underlying choice bias that is driven by value (payoff) or likelihood of occurrence (probability).

Third, we investigated whether the striatum might be part of a possible tract between vmPFC and STN. Recently, a tracing study with non-human primates showed that tracts originating in the vmPFC travel through the striatum before reaching the STN (Lehman et al. 2011). Furthermore, both the STN and the striatum are involved in the circuit underlying decision and reward processes (Frank 2009). As such, the striatum might be an important waypoint for the tract between vmPFC and STN.

## Materials and methods

### Subjects

Sixteen healthy subjects (11 female, mean age = 23.6, SD = 2.7) were included in the present study. All subjects participated in a previous functional magnetic resonance imaging (fMRI) study investigating the biasing effects of prior knowledge on choice behavior (for details, see Mulder et al. 2012). Subjects were recruited through the University of Amsterdam and had normal or corrected-to-normal vision. The procedure was approved by the ethical review board at the University of Amsterdam and informed consent was obtained from each subject. According to self-report, no subject had a history of neurological, major medical, or psychiatric disorder.

### Behavioral data

Subjects participated in two sessions of a perceptual decision-making paradigm (inside and outside the scanner environment). Since the chosen vmPFC region (see below) was identified by values of choice bias that were measured during the scanner session, we will report task details for this session only.

Subjects performed a RT version of the random-dot motion direction-discrimination task. On each trial, subjects had to decide whether a cloud of white dots moved to the left or to the right, on a black background. We matched the difficulty level of the motion stimulus across subjects at 80 % accuracy (for details see Mulder et al. 2012). Participants performed two blocks for each manipulation. Each experimental block consisted of 40 bias trials and 40 neutral trials. Each stimulus was preceded by a cue, which was an arrow pointing to the left or to the right (bias) or a square with the size of the arrow without arrow-points (neutral). In the bias trials, the cue indicated which direction had a higher likelihood of occurrence (prior probability) or which direction would lead to the highest payoff (potential payoff). In the prior probability manipulation, 80 % of the bias trials were valid (i.e., in 32 of the 40 trials the direction indicated by the cue was consistent with the direction of the stimulus) and 20 % of the bias trials were invalid (i.e., in 8 of the 40 trials, cue and stimulus direction were inconsistent). Subjects received five points for each correct response in both the biased and neutral trials. No points were given for an incorrect response. In the potential payoff manipulation, half of the bias trials were valid, i.e., cue and stimulus direction were consistent, and a large reward (eight points) was received for a correct response. On the other half of the bias trials, the cue and stimulus direction were inconsistent and subjects received two points for a correct response. No points were given for an incorrect response.

### Fitting the drift-diffusion model to the data

The DDM assumes that for two-alternative forced choice decisions, sensory evidence in favor of one of the alternatives begins to accumulate from a starting point *z* (for review, see Bogacz 2007; Gold and Shadlen 2007; Ratcliff and McKoon 2008; Wagenmakers 2009). When the evidence accumulation process reaches a threshold value, a response is initiated (Ratcliff 1978; Ratcliff and Tuerlinckx 2002; Ratcliff and McKoon 2008). The DDM can capture the effects of bias by changing the starting point (∆*z*; Edwards 1965; Laming 1968; Link and Heath 1975; Ratcliff 1985; Voss et al. 2004; Bogacz et al. 2006; Diederich and Busemeyer 2006; van Ravenzwaaij et al. 2012; Leite and Ratcliff 2011; Ratcliff et al. 1999). For neutral trials, we assumed that starting point *z* equals half the decision threshold *a*. For a valid trial, we assume that the decision process is biased by the cue toward the correct bound (*z* + ∆*z*). For invalid trials, we assume that the decision process is biased away from the correct bound (*z* − ∆*z*). We fitted the DDM to the data from each subject and each condition separately for the prior probability and potential payoff manipulations. First, for each trial-type (valid, neutral, and invalid) RT data were divided by RT-bins defined by the 10th, 30th, 50th, 70th, and 90th quantiles of the RT-distribution for correct and incorrect responses. RT-bins together with the proportion correct responses for each bin were entered in a Fortran routine that minimizes a *X* ^2^ value using a Nelder-Mead SIMPLEX optimization algorithm Ratcliff and Tuerlinckx (2002). Bias was quantified as the proportional change in the mean starting point (∆*z/z*; for details see Mulder et al. 2012). In sum, by fitting the DDM to the data we were able to capture the bias in RT and accuracy for each individual subject in a single parameter (starting point).

### Imaging acquisition

Imaging data were acquired on a 3T Philips scanner using a 32-channel head coil. For each subject, a T1 anatomical scan was acquired (T1 turbo field echo, 220 coronal slices of 1 mm, with a resolution of 1 × 1 mm, field of view = 240 × 188 × 220 mm, flip angle = 8°, TR = 8.4 ms, TE = 3.9 ms).

Four repetitions of a multi-slice spin echo (MS-SE), single shot DWI scans were acquired on a 3T MRI (TR = 7,545 ms, TE = 86 ms, 60 transverse slices of 2 mm, with a resolution of 2 × 2 mm, field of view = 224 × 224 × 60 mm). Diffusion weighting was distributed along 32 directions (b-value = 1,000 s/mm^2^). For each repetition, one image with no diffusion weighting (b0; b-value = 0 s/mm^2^) was acquired.

For the validation and visualization data set, we acquired 7T DWI data from an individual that did not participate in the current experiment. Four repetitions of a MS-SE, single shot DWI scans were acquired on a 7T MRI (TR = 5,000 ms, TE = 86 ms, 60 transverse slices of 0.87 mm, with a resolution of 0.87 × 0.9 mm, field of view = 256 × 256 × 176 mm). Diffusion weighting was distributed along 64 directions (b-value = 1,000 s/mm^2^). For each repetition, one image with no diffusion weighting (b0; b-value = 0 s/mm^2^) was acquired.

### Preprocessing

Diffusion image preprocessing was done using FSL 4.1.4 (www.fmrib.ox.ac.uk/fsl). All four DWI runs were merged and corrected for motion and eddy currents by registering each volume, including b0 volumes, to a reference DWI volume using FLIRT (Jenkinson and Smith 2001). Next, the anatomical T1 was registered to the b0 image. To use probabilistic tractography, we estimated the diffusion parameters using a sampling technique, while controlling for crossing fibers using a dual-fiber model (bedpostX; FSL 4.1.4; Behrens et al. 2007).

### Masks

The vmPFC mask was taken from a conjunction analysis between two bias manipulations reported elsewhere (Mulder et al. 2012). A sphere of 10-mm radius was drawn around the peak coordinate (MNI coordinates: 16 58-10). The STN mask was taken from the atlas probability map of the STN (Forstmann et al. 2010a, 2012; www.fmrib.ox.ac.uk/fsl; www.nitrc.org/projects/atag) and thresholded at 20 %. The striatal mask was created using subcortical regions (caudate, putamen, and nucleus accumbens) from the Harvard-Oxford subcortical structural atlas (www.fmrib.ox.ac.uk/fsl) and thresholded at 25 %. All masks were registered to each subject’s native space. First, for each subject, the MNI-standard brain was registered to the anatomical T1 image, which was already registered to the DWI image. Then, these registration parameters were used to register all masks to the individual’s native space. All registration steps were done using trilinear interpolation as implemented in FLIRT (Jenkinson and Smith 2001; FSL 4.1.4). For each individual we verified the registration procedure using visual inspection. The resulting masks had an average (SD) number of voxels of vmPFC = 484.10 (99.2), STN = 90.9 (12.2) and striatum = 2,224 (225.8).

The contralateral (exclusion) masks for the left STN and left striatum were generated using the probabilistic atlases that are implemented in FSL. For the left vmPFC we took the original peak coordinates and mirrored it to the other hemisphere by taking the negative of the *x*-coordinate. Next, a sphere was drawn around this flipped coordinate resulting in the contralateral vmPFC mask.

**Fig. 2:**
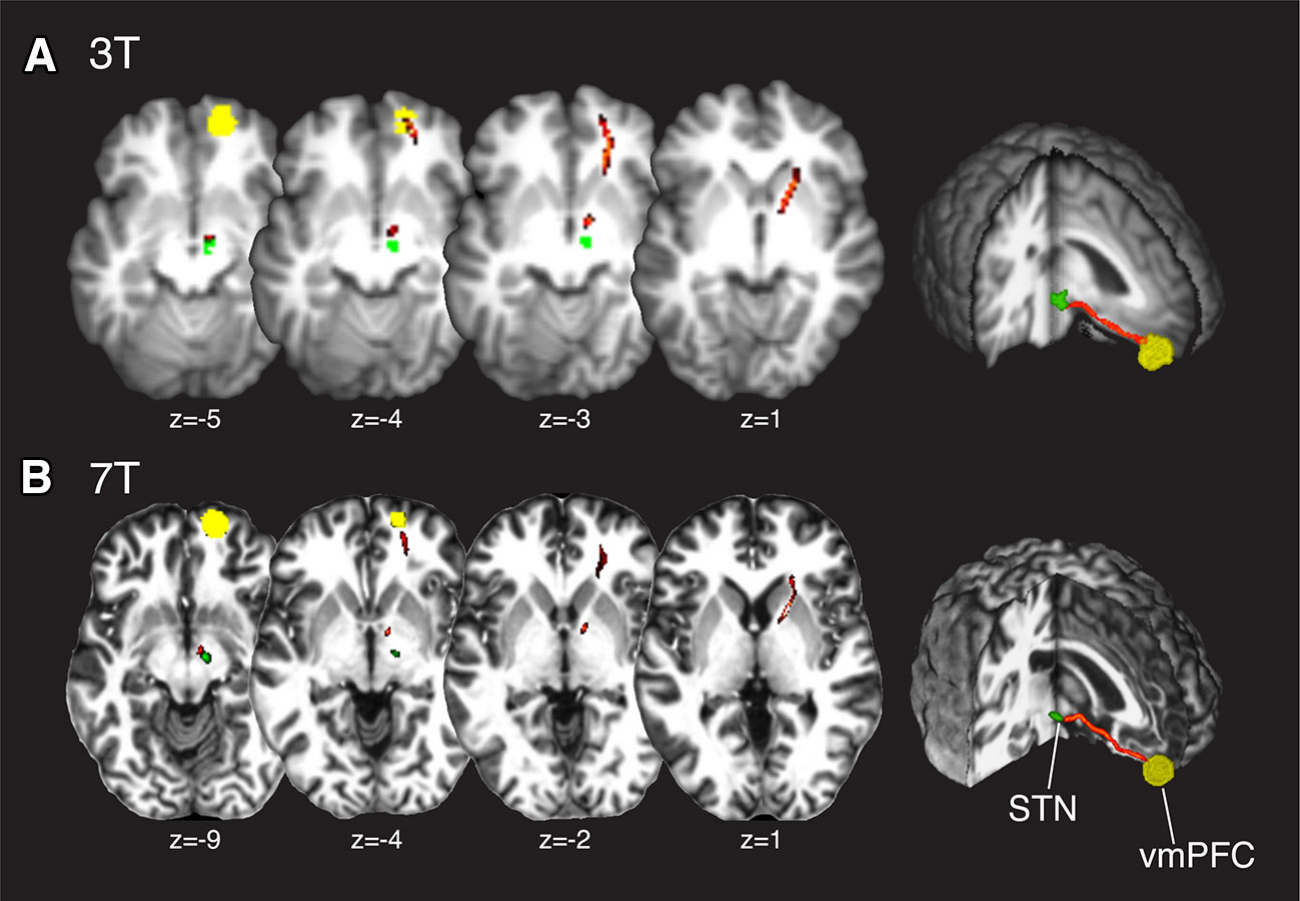
MR Images showing the connection (*red*) between vmPFC (*yellow*) and STN (*green*) for 3 Tesla (*A*) and 7 Tesla (*B)* images. Background images are anatomical scans acquired at a 3T Philips (T1) and 7T Siemens (MP2RAGE) scanner

For Fig. 2b, a manually segmented STN mask was created (Forstmann et al. 2010a, 2012) using anatomical images acquired on a 7T Magnetom MRI system (Siemens, Erlangen), with a 24-channel head array Nova coil (NOVA Medical Inc., Wilmington MA, USA). For this purpose, whole brain images were acquired with an MP2RAGE (Deichmann et al. 2000) sequence (TR = 3,000 ms, TE = 2.95 ms, TI = 1,100 ms, voxel size: 0.8 mm isotropic, flip angle = 6°, GRAPPA acceleration factor 2).

### Probabilistic tractography

Diffusion image analyses were performed using FSL 4.1.4 (www.fmrib.ox.ac.uk/fsl). Estimation of tracts was conducted using probabilistic tractography (Behrens et al. 2003a). This method uses the orientation of water diffusion within white matter fibers to reconstruct a (probabilistic) white matter tract from voxel to voxel (Mori and van Zijl 2002; Mori 2006). At each voxel, a probability distribution of the diffusion direction is estimated, resulting in probabilistic maps of fiber connectivity between brain regions. These distributions are then used to estimate the probability that a fiber starting at a voxel in a seed region, travels through any other voxel towards a voxel in a classification region, and vice versa (Behrens et al. 2003a, b).

Five thousand tracts were sampled from each voxel in the seed mask (e.g., vmPFC) at a curvature threshold of 0.2. Next, the number of samples that reach the classification target mask (e.g., STN or striatum) was measured. In addition, contralateral exclusion masks were used to discard pathways crossing over to the contralateral hemisphere before traveling to the classification target mask. To control for the difference in mask-size, the number of voxels in the seed mask for which a minimum of 10 samples reached the classification mask (Aron and Poldrack 2006; Aron et al. 2007; Forstmann et al. 2012) was divided by the total number of voxels in the seed mask. This results in a value that represents the proportion of the seed mask that was probabilistically connected to the classification mask.

As directionality cannot be inferred from tractography analyses, we followed a similar procedure in the opposite direction (where the seed and classification masks were switched). Next, probabilistic tract-strength was defined as the average of the two proportions that resulted from the seed-to-classification and classification-to-seed analyses. These values were then used in a correlational test (two-sided) together with individuals shift in starting point DDM model parameters.

### Tracts

We investigated the connection between the right vmPFC and STN while using several inclusion and exclusion masks. Below we will describe each analysis in detail.

#### vmPFC–STN

We tested for a probabilistic tract between the right vmPFC and the right STN while using the contralateral (left) vmPFC and STN regions as exclusion masks. This means that in the probabilistic tractography analyses, tracts were not allowed to travel via the left vmPFC or via the left STN, respectively.

#### Cortico-subthalamic and the striatum

To study the structural role of the striatum in the cortico-subthalamic tracts, we ran three additional probabilistic tractography analyses. First, we investigated whether the striatum served as an important waypoint for this tract. To this end, we used the mask of the striatum as a waypoint mask, which limited the probabilistic tractography analyses to only those tracts between the vmPFC and the STN that travel via the striatum. Second, an additional analysis was done to test whether a tract between the vmPFC and STN exists that does not travel via the striatum. To this end, the striatum was used as an exclusion mask, ensuring that no tracts traveled via the striatum in the probabilistic tractography analyses. Third, we investigated whether a probabilistic tract exists between vmPFC and striatum and tested for a relationship between individual differences in the probabilistic tract-strength of this tract and individual differences in choice bias.

#### Striatal-subthalamic connection

Finally, we investigated whether there was a probabilistic tract between the striatum and STN, and whether individual differences in this tract were related to individual differences in choice bias.

## Results

In this study, we seek to quantify the structural connection between the STN and a cortical region that was associated with mechanisms underlying bias in choice behavior (vmPFC). Next, we tested whether the probabilistic tract-strength of this connection was predictive for the magnitude of bias in a perceptual decision-making task. Below we first report the results of the probabilistic tractography analyses. We then report the results of the correlational analyses between individual measures of the probabilistic tract-strength and the individual measures of choice bias. In addition, we report results of probabilistic tractography analyses in which we investigated the possible role of the striatum in the cortico-subthalamic tract.

### Probabilistic tractography

For each of the 16 subjects, we estimated the probabilistic tract-strength between a functionally defined vmPFC mask and an anatomically defined STN mask using probabilistic tractography (Behrens et al. 2003a). The results show a probabilistic tract [mean (std) tract − strength = 0.30 (0.26)] starting from the vmPFC traveling via the anterior corona radiata (acr) and the anterior limb of the internal capsule (aic) through the STN. The tract is depicted for one subject in Fig. 2a.

Since the registration of the probabilistic STN mask to individual space might induce inter-individual variability in the size and localization of the STN, we validate the analysis on an independent DWI data set that was acquired on a Siemens 7T Magnetom MRI system. Instead of using the probabilistic STN map, we used a mask of the STN that was manually segmented for this participant (see Forstmann et al. 2010a, 2012 for segmentation procedure details). Note also that this subject did not participate in the current 3T study, and as such was not part of the analyses described below. Results are shown in Fig. 2b.

### Individual differences in probabilistic tract-strength and choice bias

All 16 healthy subjects performed an RT version of a random-dot motion direction-discrimination task in which bias was manipulated by a cue indicating a higher value associated with the motion stimulus (potential payoff) or a higher probability of the direction of the motion stimulus (prior probability). Subjects tended to make more and faster decisions toward the alternative that had the largest value or was the most likely. Bias was quantified by fitting the drift-diffusion model to the behavioral data. The results show that the biasing effect was primarily driven by a change in the starting point of the accumulation process (Mulder et al. 2012). These individual differences in starting point were used to test whether the vmPFC–STN connection is associated with mechanisms underlying choice bias that is driven by value (payoff) or likelihood of occurrence (probability).

**Fig. 3:**
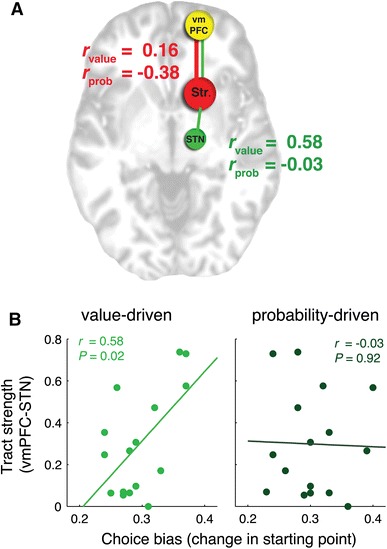
**a** Schematic representation of tracts between vmPFC, striatum, and STN. No significant correlations were found between vmPFC–striatum tracts (*red*) and choice bias. For the vmPFC–STN connection (*green*), we found that individual differences in probabilistic tract-strength predict individual differences in value-driven but not probability-driven choice bias (**b**)

We performed a two-sided correlational test using the individual values of the connection strength between vmPFC and STN together with the individual values of choice bias (e.g., shift in starting point of the accumulation process). Results show that the strength of the connection between vmPFC and STN were associated with the shift in starting point for the potential payoff manipulation (*r* = 0.58, *P* = 0.02), but not for the prior probability manipulation (*r* = −0.03, *P* = 0.92; see Fig. 3). These results suggest that the connection strength is predictive of choice bias, but only when it is value-driven.

To test whether the probabilistic tract-strength was a better predictor for value-driven compared to probability-driven choice bias, we performed a permutation test. At each of ten thousand permutations we randomly reordered the tract-strength values, and re-paired the new values with the choice bias values. We recalculated the explained variance (*r* ^*2*^) for each manipulation and calculated the difference between them. This resulted in a distribution of all possible differences under the null hypothesis that there is no difference between the explained variances of both manipulations. Next, a two-sided *p* value was calculated from the proportion of times in which the (absolute) difference computed from the permuted values was greater than the (absolute) difference between original values. Results confirmed that probabilistic tract-strength was a better predictor for value-driven choice bias compared to probability-driven choice bias (*P* < 0.05).

### Cortico-subthalamic and the striatum

Our results show that tracts originating in the vmPFC travel through the striatum before reaching the STN (Fig. 2), which is in line with findings from tracing studies with non-human primates (Lehman et al. 2011). Furthermore, the cortico-striatal circuit is associated with the adaptation of the decision threshold for choices under speed stress (Forstmann et al. 2008, 2010a; van Maanen et al. 2011). To investigate whether the relationship between the strength of the vmPFC–STN tract and choice bias depends on the striatum, we re-ran the probabilistic tractography analyses with an anatomically defined (probabilistic) mask of the striatum (see “Methods”). This mask was first registered to each individual’s native space and then used as waypoint, exclusion or classification mask in the following probabilistic tractography analyses:

1. *Striatum as a waypoint mask*: In this analysis, tracts were required to pass through the striatum. Tracts that did not pass the striatum were discarded from the tract-strength calculation. Probabilistic tract-strength did not change [mean (SD) tract-strength = 0.30 (0.26)] and the relationship with choice bias was as strong as before (*r* = 0.58, *P* = 0.02).
2. *Striatum as an exclusion mask*: In this analysis, all pathways were discarded if they enter the striatum. No tracts arrived at the STN [mean (SD) tract-strength = 0.0 (0.0)].
3. *Striatum as a target mask*: Here we tested for a probabilistic tract between vmPFC and striatum, while excluding those tracts that might travel via the STN. We found a tract between vmPFC and striatum [mean (SD) tract-strength = 0.71 (0.11)]. No significant relationship was found between the strength of the vmPFC–striatum tract and choice bias (*r* = 0.16, *P* = 0.56 for potential payoff and *r* = −0.38, *P* = 0.15 for prior probability).

### Striatal-subthalamic connection

Although the individual differences in cortico-striatal connection strength could not explain the individual differences in choice bias, it might be the case that the relation is driven by a connection between the striatum and the STN. To test this, we run the probabilistic tractography analyses between the striatal and STN masks. Results show a connection between the striatum and STN [mean (SD) tract-strength = 0.74[0.09]). No significant relationship with choice bias was found (*r* = 0.05, *P* = 0.86 for potential payoff and *r* = 0.19, *P* = 0.49 for prior probability).

Note that the strength of the probabilistic tract for the different parts (vmPFC–striatum and striatum–STN tracts) deviates from the strength of the entire tract (vmPFC–STN). This is inherent to the method: Although the probabilistic tractography will ‘follow’ the existing white matter tracts (see also Jbabdi et al. 2013), it does not represent an absolute measure of white matter volume (for further details see Johansen-Berg and Behrens 2009). Rather, it represents the likelihood that a tract will travel from a seed to a target region, which will vary with the distance between the seed and target regions. As such, the probabilistic strength of a tract will likely deviate from the strength of its parts.

In sum, the results show a structural connection between vmPFC and STN that travels through the striatum. As such, it is likely that the striatum plays a role in the relation between the probabilistic strength of the vmPFC–STN tract and choice bias. However, no correlation was found between the strength of one of the two parts of the tract (vmPFC–striatum or striatum–STN), suggesting that the relationship was not driven by one of these parts alone.

## Discussion

In this study, we identified a structural tract in vivo between the STN and a cortical region that was associated with mechanisms underlying bias in choice behavior (vmPFC). We used (DWI) and probabilistic tractography to measure the tract-strength and tested whether individual differences in this tract-strength were predictive for individual differences in the magnitude of choice bias. Choice bias was quantified using the DDM where a shift in the starting point of evidence accumulation produces faster choices and a larger proportion of choices for an alternative that is more likely or more valuable. Results show that the STN is structurally connected with this region of the vmPFC and that the strength of this connection is predictive for choice bias towards an alternative that is more valuable, but not for choice bias towards an alternative that is more likely.

Recently, we showed that the vmPFC was active for both value-driven and probability-driven choice bias. This raises the question why the individual differences in the strength of the cortico-subthalamic tract would be selectively related to value-driven, but not to probability-driven choice bias. Note that the absence of a relationship between the probabilistic tract-strength and probability-driven choice bias does not have to imply that the connection is not used in probability-driven decision processes at all. Rather, it might be the case that individual differences in processing the information about the likelihood of an alternative cannot be explained solely by the differences in strength of the vmPFC–STN tract. Indeed, activation patterns related to probability-driven choice bias show a broadly distributed network of brain regions (Mulder et al. 2012; Forstmann et al. 2010b; Preuschhof et al. 2010), whereas the network for value-driven bias might be less diffuse (Mulder et al. 2012). A possible explanation may involve the type of prior information that is processed before the moment of choice; although the consequences might be the same (a shift in starting point), information about the possible direction of the stimulus might rely on other cognitive resources than information about the value associated with the alternatives. It might be the case that the latter is more closely related to the reward-circuit, and as such is more sensitive to individual changes therein.

A key question remains: how does the STN use the value-based information to bias the decision process? One hypothesis is that the STN has to resolve the conflict between the possible choice outcomes that compete for access to the motor system (Zaghloul et al. 2012). According to this idea, the value-driven bias signal causes a difference in conflict between the alternatives. First, the STN pre-selects the motor program that is associated with the most valuable alternative. This pre-selection is then put on a hold, until an action has been chosen in response to the stimulus. When the appropriate motor response to the stimulus is not compatible with the pre-selected program, the conflict between prior (cue) and actual information (stimulus) is high, causing the STN to respond and inhibit the motor system (Gurney et al. 2001; Humphries et al. 2006; Zaghloul et al. 2012; Frank 2006). As such, a choice with a preceding invalid cue (that is, representing prior knowledge that is not compatible with the stimulus) would cause slower and fewer correct decisions. In contrast, when the pre-selected motor program is compatible with the upcoming stimulus (valid), the conflict between the pre-selected (cue) and selected (stimulus) responses will be low, resulting in faster and more correct decisions.

Such a scenario would be in line with the behavioral expectations of the DDM, where a shift in the starting point of the accumulation process results in faster response times for valid but slower response times for invalid preceding cues (e.g., van Ravenzwaaij et al. 2012; Mulder et al. 2012; Edwards 1965; Laming 1968; Link and Heath 1975; Ratcliff 1985; Voss et al. 2004; Bogacz et al. 2006; Leite and Ratcliff 2011; Ratcliff et al. 1999; Diederich and Busemeyer 2006). Within this framework, the mechanisms underlying choice bias might be similar to an adaptation of the decision threshold. When two alternatives are highly conflicting, an increase in the decision threshold results in a larger distance between the starting and ending point of the accumulation process, which in turn results in a longer time to decide, reducing premature choices (Frank 2006). Similarly, a shift in the starting point towards the preferred alternative (i.e., low conflict) results in a smaller distance towards the preferred alternative, with faster and more preferred choices as a consequence (choice bias). As such, the model-based framework provides a possible explanation of how the STN might use the value-driven information in biasing perceptual choices.

A second remaining open question is: what is the role of the striatum in the relationship between the probabilistic tract-strength and choice bias? Our data shows that the vmPFC–STN tract travels through the striatum. This is in line with animal studies showing projections from the medial/orbito frontal regions to the STN via striatal regions (Lehman et al. 2011; Maurice et al. 1998, 1999). As noted earlier, the strength of the probabilistic tracts between the striatum and vmPFC or STN (vmPFC–striatum and striatum–STN) were different from the strength of the entire tract (vmPFC–STN). Although this is inherent to the method, individual differences in tract strength for the whole tract might still be similar to the individual differences in tract strength of its parts, meaning that the correlation between tract-strength and behavior might still be apparent for the vmPFC–striatum and striatum–STN tracts. However, the relationship with choice bias could not be explained by the cortico-striatal or striatal-subthalamic connections. Possibly, both the cortico-striatal and cortico-subthalamic circuits process information concurrently in the reward and motor domain (Nambu et al. 2002; Temel et al. 2005; Teagarden and Rebec 2007; Heida et al. 2008).

Earlier findings show that the striatum plays an important role in adjusting the decision threshold under speed stress (Bogacz et al. 2010b; Frank 2006; Forstmann et al. 2008, 2010a; van Maanen et al. 2011). According to these studies, people lower their decision threshold by activating the striatum, resulting in a release of inhibition of selected motor programs with faster choices (and more errors) as a consequence (speed–accuracy tradeoff; Bogacz et al. 2010b). Interestingly, the stronger the connection strength between the striatum and pre-supplementary motor area (pre-SMA), the more flexible subjects are in changing the decision threshold (Forstmann et al. 2010a). This might suggest that the cortico-striatal connections are involved in an increasing speed, without considering which response is the most appropriate one. In contrast, activating the STN results in an inhibition of a motor response, preventing a premature response to occur before the appropriate one is actually chosen (Frank 2006). Along these lines, the vmPFC–STN tract might have a purpose in mediating the selection of the most valuable option, and inhibiting the least appropriate one.

Taken together, in this study we quantified a structural connection between vmPFC and STN using probabilistic tractography. We showed that the strength of this connection predicts the magnitude of value-driven choice bias in a simple perceptual decision-making task. These results confirm the importance of cortico-subthalamic structural networks in perceptual decision making.

## Footnotes

1 This region was labeled as medFG in Mulder et al. (2012). However, peak coordinates are in accordance with the ventral part of the medFG (MNI coordinates: 16 58–10; BA11).
